# Supplementary material for: Chromothripsis during telomere crisis is independent of NHEJ, and consistent with a replicative origin
Source: Genome Res. 2019 May;29(5):737–49. doi: 10.1101/gr.240705.118 (PMC6499312; doi:10.1101/gr.240705.118)
Supplement: Supplemental Material [file supp_gr.240705.118_Supplemental_file_1.zip › contigs/annotated_contigs/DB91/contig.2.DB91_length_606_mean_cov_12.6237623762.docx]

**DB91_length_606_mean_cov_12.6237623762**

```
GGTCAGGGCTGCAAATGGGAACAAAGTAAAGCTTTTGGCAGTTTATTCTGTCAAAAATATTGCAAATGCTGTTGTAAATTAAAAAGCTC
 >chr5:77427893-77428223 - E=7e-184 p=5e-02
AACTTTGAAGATAAAACTGTTTTTGATGAAAGGACGTTGTCATGGTAAAAATCAATGTTCTTATTTAATTAAGAAAACTATGGAAGAGA

AATGTTTTAGGAATTGGTTGTTGTGCATGCGAACAAACTGCTTATTCAGCCAGTTGAAATAAAACCGTAGTTGTCACATTTACTGATAT

TAGTATACACAGTTTGACCGAACTATAAAATTCTTGTGATGAAACTGATGTTGAAAAACCA|TT|TATTTTAGTTGCAGATTTAAAATC
                                                              >chr5:77426389-77426667 -
TCAGCACTCAAATGGACTTTAGAGTTAACCAAGTTGTAAATTCTAACTCCACATTTGAGAAAACTAATGATTAAGTGGATTTCCCAGGT
 E=1e-155
TTTTCCAGCAAATTAATTACATGGTTTAGTGTACCACATTTTCTTTTAAGTTGGTTTTGAATTGTATTCCTCATTTGGTGAATACTTTG

CAAATTGTCTTAAAAAATGAATCATAGAAATTTTCTTACCTGTACTTGAACCAGCATGGTCTAGCAATTAGTGA
```
